# Conceptualising the experiences of continuing professional development of young private sector audiologists as an attribute of andragogy

**DOI:** 10.4102/hsag.v29i0.2683

**Published:** 2024-07-05

**Authors:** Suvishka Barath, Andrew J. Ross

**Affiliations:** 1 Department of Family Medicine, College of Health Sciences, University of KwaZulu-Natal, Durban, South Africa

**Keywords:** audiologist, continuing professional development, education, knowledge, adult learning theory, healthcare, private sector, South Africa

## Abstract

**Background:**

Continuing professional development (CPD) is an ongoing learning process that builds on initial training and education to improve competency. Low compliance rates of audiologists adhering to CPD have been reported by the Health Professions Council of South Africa. However, there is an absence of research on the uptake of CPD from the perspective of young audiologists working in the private sector.

**Aim:**

This study aimed to explore the experiences and views of young audiologists working in the private sector on continuing professional development.

**Setting:**

The study was conducted in KwaZulu-Natal province, South Africa.

**Methods:**

The descriptive, qualitative approach entailed conducting 11 online, semi-structured interviews with audiologists working in the private sector. Semi-structured interviews consisted of open-ended questions, and the qualitative data were thematically analysed. The adult learning theory, andragogy, was used as both the conceptual and analytical framework.

**Results:**

Five andragogy concepts were used to analyse the data, with eight sub-themes emerging related to: self-concept, adult learning experiences, readiness to learn, orientation to learning and internal motivation.

**Conclusion:**

The experiences of audiologists in the private sector on CPD aligned with the concepts of andragogy. Audiologists’ experiences need to be taken into consideration during the planning and implementation of CPD for it to be relevant, effective and purposeful.

**Contribution:**

This study highlighted the experiences of audiologists on CPD working in the private sector with continuing professional development.

## Introduction

Continuing professional development (CPD) aims to improve healthcare professionals’ clinical practice by updating their knowledge, skills and ethical attitudes to keep abreast with the best practices (Health Professions Council of South Africa [HPCSA] 2021). However, the literature provides evidence that CPD is underutilised by healthcare professionals to close the gap in knowledge and improve the quality of care provided by clinicians, including audiologists (Davis & McMahon 2018).

As a result of the advances in science and technology, as well as rising patient demands and needs, healthcare systems are continually changing (Hakvoort et al. 2022). A synthesis of existing research highlights that the unique dynamics of knowledge and skills acquired during training is not being applied in clinical practice, also known as ‘the knowledge-to-action gap’ (Hakvoort et al. 2022). Closing this gap requires a ‘transfer of knowledge’ that enables healthcare professionals to apply newly acquired knowledge and skills. However, access to knowledge and experience is often determined by hierarchy and cultural practices in the work environment, which vary in the educational activities they offer and the degree of job autonomy they allow (Hakvoort et al. 2022).

In South Africa, audiology has grown over the past five decades from a combined profession of speech and hearing therapy into the two related but distinct professions of speech-language therapy and audiology (Breytenbach, Kritzinger & & Soer 2015). Audiologists have access to a variety of practicing options in various work environments in South Africa once they complete their year of public sector community services, with some going into private practice (Breytenbach et al. 2015). This study focuses on the private sector, where audiologists render services in independent clinics, practices or medical facilities that are not connected to government organisations or publicly sponsored healthcare systems (Kochkin 2009).

In private practice, audiologists typically have a broad scope of practice that is focused on the prevention, identification, diagnosis and evidence-based intervention and treatment of hearing, balance and other related disorders across all age groups (American Speech-Language-Hearing Association [ASHA] 2018). These services include, but are not limited to, (1) standard behavioural diagnostic audiological assessments; (2) electrophysiological assessments (otoacoustic emissions [OAEs], auditory brainstem response [ABR] and auditory steady-state response [ASSR]); (3) ototoxicity monitoring; (4) rehabilitation technology (e.g. hearing aid evaluations, fittings and adjustments and cochlear mapping); (5) vestibular assessments and rehabilitation; (6) auditory processing disorder evaluations; (7) tinnitus management; (8) wax management; (9) aural rehabilitation and (10) occupational audiology (hearing conservation and preservation) (ASHA Association 2018). This is in addition to the skills needed to run a business, which includes issues related to financial and tax management as well as labour legislation.

The HPCSA is the statutory body that oversees the education, training and registration of health professionals practicing in South Africa, including audiologists. The HPCSA requires health professionals to be CPD compliant as a prerequisite for annual re-registration for independent practitioners to be able to practice within South Africa (HPCSA 2021). Continuing professional development activities are accredited by the HPCSA, each activity being allocated a number of continuing education units (CEUs), which refers to the value of the learning activity (HPCSA 2021). Healthcare professionals are required to accumulate points each year, with specific numbers contributing towards clinical practice, ethics, human rights and health law (HPCSA 2021). Continuing education units are valid for 12 months from the date the activity commenced, audiologists being required to obtain at least 30 CEUs annually, with a minimum of 5 for ethics, human rights and health laws (HPCSA 2021).

Audiologists within the private sector engage in a variety of CPD activities that include conferences, workshops, seminars and/or webinars, online courses, online journal articles and simulation training. Information regarding the details of CPD events is generally shared (1) on digital platforms and/or social networking sites, such as LinkedIn; (2) on mobile instant message (MIM) platforms, the most common being WhatsApp groups and (3) via the South African Speech-Language-Hearing Association website, which is the professional body they are affiliated to (Pimmer, Lee & Mwaikambo 2018).

As per the various registration categories of HPCSA, audiologists fall under the speech, language and hearing (SLH) board, the overall CPD compliance rate of all those registered with this board being 37.2% as of January 2024 (HPCSA 2024). Factors contributing to this low rate of CPD attendance among audiologists are not well understood, making it important to understand their knowledge and experience related to engaging in these activities. Although there is considerable literature about CPD programmes and their design process globally, little has been reported from the perspective of audiologists, particularly those in private practice.

The conceptual framework employed in this study is aligned with the learning theory for adults by Malcolm Knowles (1985), also known as andragogy, with relevant concepts being used for as an analytical framework. Andragogy is based upon five concepts (Knowles 1985; Magwenya & Ross 2021; VanNieuwenborg et al. 2016) of self-concept, adult learning experience, readiness to learn, orientation to learning and internal motivation:

- Self-concept: adults are autonomous and like to exercise control over the techniques and goals within the learning that is occurring, with self-directed learning being appropriate.
- Adult learning experience: adults have a wealth of experiences and knowledge and learn new things by drawing on their prior experiences.
- Readiness to learn: adults are relevancy oriented and find it easier to learn when there is a reason to acquire new knowledge.
- Orientation to learning: adults are practical and learn best when knowledge is presented in a realistic context that is applicable to their own practice.
- Internal motivation: adults demonstrate high levels of motivation to learn when new information helps them solve significant problems or contributes to career advancement.

It is important to understand the reasons that determine why audiologists participate in CPD activities, as this would assist in creating relevant programmes, improving participation and hopefully encouraging a change in behaviour and clinical practice (Magwenya & Ross 2021). This may also help the HPCSA to review the current CPD programme and may contribute towards the development of a new system that ensures that audiologists take responsibility for their own CPD. In the absence of knowing what promotes and hinders audiologists’ participation in their own professional development, it is not possible to develop a system from which they will benefit, making it important to establish their perspective on CPD. The aim of this study was to explore the CPD experiences of young audiologists who are working in the private sector in KwaZulu-Natal (KZN), South Africa.

## Research methods and design

### Study design

A descriptive, qualitative research approach was employed as it was an appropriate methodology to explore audiologists’ individual experiences of CPD within the context of the private sector.

### Setting

The research took place in KZN province, which has the highest number of audiologists practicing in South Africa with 27.8% practicing in the public sector and 72.2% in the private sector (Pillay et al. 2020).

### Study population and sampling strategy

Purposive sampling was undertaken by circulating a participant recruitment poster throughout the KZN on various social media platforms that mainly consisted of audiologists, with those being interested in participating being requested to contact the researcher. Snowballing sampling was then used to obtain referrals to other audiologists, with all those who responded positively being provided with an information document via email or WhatsApp to help them reach an informed decision. Those who met the inclusion criteria and were willing to participate were provided with informed consent forms before the interviews. The inclusion criteria included audiologists practicing in the private sector in KZN registered with the HPCSA as independent practitioners. Audiologists were also required to have previous CPD experience and had graduated between 2017 and 2022. Practitioners were excluded if they were dually qualified as speech-language therapists and audiologists, as this may confound the results and were practicing in the public and private sectors simultaneously.

Eleven audiologists were interviewed individually, as data saturation was obtained.

### Data collection

Data were collected during November and December 2023 once ethical approval from the University of KZN’s Human and Social Sciences Ethics Committee had been obtained. The interviews were conducted online using WhatsApp voice calls and recorded with permission from the participants, with English being the preferred language of all the participants. A semi-structured interview guide was utilised to conduct the interviews, the opening question being ‘Tell me about your experiences of CPD and why you participate in CPD activities’. The interview guide contained mainly open-ended questions that covered issues such as what CPD activities they participated in, how often, reasons for participating, value of the CPD activity and probing questions that prompted further discussion. The interviews lasted between 20 and 40 min.

### Data analysis

After data collection, the audio-recorded interviews were transcribed verbatim and deductively analysed using Braun and Clarke’s (2006) six steps of thematic analysis process with respect to the five concepts of the andragogy theory. Using NVivo, a qualitative data analysis software (QDAS), analysis was guided by key concepts from the andragogy framework. The data analysis was ongoing during the data collection process and after 11 interviews no new themes emerged.

### Reliability and validity

A pilot study was conducted with two participants to ensure the credibility and efficiency of the data collection tool. Credibility was also achieved, as the research questions were derived from the themes based on a comprehensive literature review. Recognising that the researcher contributes to the research process is a component of reflexivity; an independent qualitative coder was engaged to increase data trustworthiness and decrease bias. After independently identifying codes, categories and themes, a consensus meeting took place where they discussed and agreed on codes and themes.

### Ethical consideration

Ethical approval was obtained from the University of KZN’s Humanities and Social Sciences Ethics Committee (HSSREC/00006281/2023). Privacy and confidentiality were maintained for all participants of this study, with no names or information related to the private practice’s workplace being disclosed in the study.

## Results

### Description of participants

Of the 11 participants, 8 were females and their ages ranged from 23 to 26 years, with the duration of working within the private sector being 1–5 years. Their practice profiles covered a range of areas, with their CPD activities mainly consisting of attending workshops and accessing online journal articles (Table 1).

**TABLE 1:** Description of participants.

Participant no.	Age (years)	Gender	Education institution	Years in private practice	Practice profile	Type of CPD activity undertaken
1	23	Female	UKZN	1	Standard adult and paediatric behavioural diagnostic assessment Electrophysiological assessments	Online journal articles In-person workshops
2	23	Male	UKZN	1	Standard adult behavioural diagnostic assessments Hearing aid evaluation, fitting and adjustments Adult aural rehabilitation	Online journal articles Webinars Online training courses
3	24	Female	UKZN	1	Standard adult behavioural diagnostic assessment Hearing aid evaluation, fitting and adjustments Adult aural rehabilitation	Online journal articles Webinars
4	23	Female	UKZN	1	Hearing screening Standard adult behavioural diagnostic assessments Hearing aid evaluations, fitting and adjustments Adult aural rehabilitation	Online journal articles Webinars
5	25	Female	UKZN	2	Hearing screening Standard adult behavioural diagnostic assessments Hearing protection devices Hearing aid evaluations, fitting and adjustments Adult aural rehabilitation	Online journal articles Webinars In-person courses
6	25	Female	UKZN	2	Hearing screening Standard adult behavioural diagnostic assessment Hearing aid evaluation, fitting and adjustment Aural rehabilitation	Online journal articles
7	26	Male	UKZN	2	Hearing screening Standard adult behavioural diagnostic assessment Ototoxicity monitoring Occupational audiology Wax management Hearing aid evaluation, fitting and adjustment Aural rehabilitation	Online articles In-person workshops
8	26	Male	UKZN	2	Standard adult and paediatric behavioural diagnostic assessment Electrophysiology assessments Vestibular assessments Wax management Hearing aid evaluation, fitting and adjustments	Online journal articles Webinars Online training courses
9	26	Female	UKZN	5	Standard adult behavioural diagnostic assessment Wax management Ototoxic monitoring Tinnitus management Speech mapping and/or real ear measurements Hearing protection devices Hearing aid evaluation, fitting and adjustment	In-person workshops Online journal articles Online training courses
10	24	Female	UKZN	2	Standard adult behavioural diagnostic assessment Hearing aid evaluation, fitting and adjustments Aural rehabilitation	Online journal articles In-person workshops
11	23	Female	UKZN	1	Hearing screening Standard adult and paediatric behavioural diagnostic assessments Hearing aid evaluation, fitting and adjustment Wax management Aural rehabilitation Community outreach	Online journal articles

CPD, continuing professional development; UKZN, University of KwaZulu-Natal.

## Results

**FIGURE 1:**
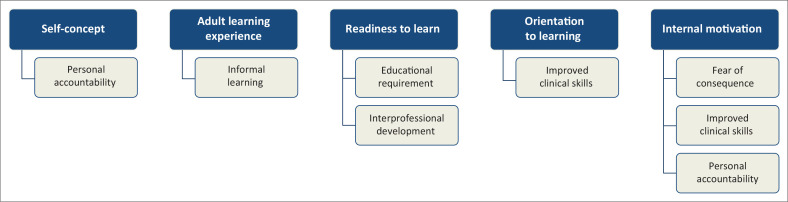
Themes and subthemes that emerged in line with andragogy.

Five themes and eight subthemes were identified from the data in line with the concepts of andragogy (Figure 1).

### Theme 1: Self-concept

Autonomous adults like to exercise control over the goals within which learning is taking place. One subtheme emerged, which related to participants acknowledging their own responsibility for improving their knowledge and skills and accepting that they were accountable for *upskilling yourself*.

#### Subtheme 1.1: Personal accountability

The participants acknowledged that this was something that they needed to take responsibility for, and that taking part in different CPD activities required a disciplined approach to their professional development. They enjoyed the fact that they had to read widely and engage with articles and/or discussions to obtain CPD points, which helped them to feel more confident in that specific area:

‘I would say that’s the first reason, but also self-growth as a professional in terms of upskilling yourself in areas where you feel like you not confident, obviously you’re gonna go and read up on those area, you’re gonna attend courses, you going to watch online courses also. So, it’s a matter of developing yourself as a professional.’ (Participant 7, 26, Male, UKZN)

### Theme 2: Adult learning experience

Adult learners are not a ‘blank page’ but have a wealth of experiences and knowledge based on prior experiences and learn new things by drawing on these experiences, with one subtheme emerging related to informal learning.

#### Subtheme 2.1: Informal learning

Participants noted that when attending CPD events there were opportunities to socialise and network with their peers, share their own experiences and reflect and learn from their colleague’s experiences. They recognised the wealth of experiences that other audiologists had, and by engaging with them informally at CPD meetings and discussing challenging cases, they were able to learn new things. The CPD activities provided them with the platform to seek out diverse learning experiences that aligned with their professional development needs. This informal learning was often perceived to be more beneficial than the formal learning that was provided:

‘I feel like you meet so many people and you learn so much just from the courses and from other audiologists.’ (Participant 11, 23, Female, UKZN)

‘You can discuss strategies, new things that you’ve come across, new skills that you’ve learnt and its easier to share struggles that you experiencing as an audiologist.’ (Participant 6, 25, Female, UKZN)

### Theme 3: Readiness to learn

A readiness to learn is an important prerequisite to adults learning, with participants indicating that they attended CPD activities because they were eager to learn about advances in the profession and establish if they could be applied to their practices. Two subthemes emerged: educational requirements and interprofessional development.

#### Subtheme 3.1: Educational requirement

Participants indicated that as audiology is:

‘[*A*]n ever-growing field.’ (Participant 2, 23, Male, UKZN)

They felt the need to address their knowledge gaps and stay abreast of the latest advancements by participating in CPD activities as:

‘[*I*]t’s important to keep ourselves updated with the trends in the profession.’ (Participant 2, 23, Male, UKZN)

They stated that their desire to participate in CPD was influenced by or in response to a particular clinical case they were handling or had previously handled and that they wanted to gain more knowledge about managing similar situations. The relevance of the CPD activity to their daily work made it easy for them to participate and apply the learning:

‘You basically build knowledge on that so you can relate whatever new or recent research that you’ve read, or the seminar you attended or article you read, you can relate it to the case and it basically can increase your outlook on things when you deal with certain cases. Yeah, and also, maybe you read it from somebody else’s perspective, so you have a different outlook when you deal with that certain case.’ (Participant 4, 23, Female, UKZN)

Some indicated that without a readiness to learn and a recognition of the need to learn, learning does not occur. To stay up-to-date and provide the service of excellence that was expected of them, audiologists needed to engage in CPD activities. Audiology is a growing field, and with new developments in technology, they felt motivated to participate in CPD activities. Engaging in CPD helped them to holistically improve patient care as they developed a more comprehensive view of the different aspects of healthcare that they could provide:

‘Our profession audiology is an ever what you call growing field so it’s important to keep ourselves updated with the trends in the professions.’ (Participant 2, 23, Male, UKZN)

‘If there are tips that I can use to broaden my knowledge to give patients better treatment and to improve their overall health and wellness, why not?’ (Participant 10, 24, Female, UKZN)

#### Subtheme 3.2: Interprofessional development

Physiotherapists, occupational therapists, psychologists and other healthcare professionals participated in some of the CPD activities that the participants attended. Participants noted that exposure to different perspectives and skills relevant to the overall health sector enriched their learning experience. Attending CPD events with other healthcare professionals provided them with the opportunity to broaden their knowledge in all facets of healthcare, in addition to providing evidence-based practice in their field:

‘I would say that there are activities that do contribute, especially when it has to do with research affecting other healthcare conditions and hearing, like new research coming up about dementia and hearing loss, diabetes and hearing loss. All of that definitely impacts the way you carry out work with your patients, because now when you advise them you’re able to say that this research study found this, this and this.’ (Participant 6, 25, Female, UKZN)

‘Engaging in these CPD activities help in growing our medical knowledge.’ (Participant 9, 26, Female, UKZN)

### Theme 4: Orientation to learning

Andragogy highlights that adults are practical and learn best when knowledge is presented in a realistic context and is applicable to their practice. One subtheme emerged related to this aspect, that being improved clinical skills.

#### Subtheme 4.1: Improved clinical skills

Participants acknowledged that their learning was enhanced when the CPD content was directly applicable to their practice and helped them to strengthen their skill set. The applicability of the material facilitated the transfer of theoretical knowledge acquired from the CPD activities into practical evidence-based practice. This helped to orientate them towards their learning goals and provided clarity on what they needed to focus on:

‘[*CPD*] … reminds me of some stuff and it helps me to improve whatever I do, because whenever you read something, you don’t just read and forget about it, you keep it in you and try and apply it practically.’ (Participant 7, 26, Male, UKZN)

Continuing professional development provided an avenue for them to refine their skills, which resulted in increasing their confidence within a specific area by acquiring new knowledge and building on their existing skills:

‘I think to learn more and also improve my existing skills of stuff that I’m not confident in so that I can work better and understand better.’ (Participant 1, 23, Female, UKZN)

### Theme 5: Internal motivation

Internal motivation is a key component of adult learning, as adults demonstrate high levels of motivation to learn when new information helps them solve significant problems or contributes to career advancement. Three subthemes emerged under this concept: fear of consequences, improved clinical practice and personal accountability.

#### Subtheme 5.1: Fear of consequence

Despite acknowledging the importance of CPD for personal accountability, an important intrinsic motivation for their participation was (often) the statutory CPD requirement of the HPCSA, and the fear of not meeting the CPD requirement would prevent them from re-registering, which would impact on their ability to work as an audiologist:

‘There’s no other way for me to engage with the CPD, I always engage myself with the CPD’s just for the points, other than that I wouldn’t be doing any articles. I wouldn’t be even interested, because I feel like it’s a waste of time, because if I read through the articles, there’s no change to my professional practice, so it’s [*only*] a matter of me obtaining the points.’ (Participant 3, 24, Female, UKZN)

One participant stated that her understanding of HPCSA’s directives was her driving force to adhere to CPD requirements, as failure to do had negative consequences for her registration status:

‘[*D*]efinitely to get the CPD points you need it in order to keep practicing in Audiology, so according to the Health Care Professions Council, you need a certain amount every year for you to continue practice, because if you don’t, you have to take a board exam or you could be departing from the counsel.’ (Participant 4, 23, Female, UKZN)

#### Subtheme 5.2: Improved clinical practice

All the participants indicated that improved patient care is important, and that continuing knowledge acquisition was essential for allowing them to provide services within the private sector that are in line with the best clinical and ethical practices:

‘I work in a private practice that deals a lot with hearing aids, and we get a lot of these CPD activities that focuses on hearing aids, real ear measures, programing, verification, so I keep myself updated, because whatever I learn in those activities I carry over to the practice, and it not only benefits me it also benefits people I see.’ (Participant 2, 23, Male, UKZN)

‘[*B*]y doing CPD, we continuously improve our skills and our knowledge about what’s being put out in terms of research and best practice, the best ethical procedures to follow. So, it’s very important with patient care, because we’re dealing with people’s lives and it’s important to give them the best that you know you can.’ (Participant 6, 25, Female, UKZN)

#### Subtheme 5.3: Personal accountability

Personal accountability was also an important internal motivation to participate in CPD activities, as participants felt accountable for the services they provide and wanted to keep up-to-date and provide the best services possible, which regular CPD activities helped them to achieve. They further highlighted that patients seen in private practice expect professionals to offer the best evidence-based practice, resulting in them feeling that it was their professional obligation to participate in CPD to improve the outcome of the services they provide, as well as to ensure job satisfaction:

‘It also helps me increase and improve my knowledge, because now especially, when you are in private practice, you have to keep up to date with a lot of things going on in audiology. I mean, if a patient comes to you and asks to you about certain stuff, you need to know because you provide this … private practice is so different, they are paying so you have to, you know, to the best of your ability, know what’s going on in audiology at all times.’ (Participant 5, 25, Female, UKZN)

## Discussion

The aim of this study was to explore the experiences of audiologists in private practice in KZN with respect to CPD and is possibly the first to do so in South Africa, particularly within the private sector.

Participants acknowledged that CPD is their own responsibility related to their ongoing learning, both formal and informal, which results in the acquisition of knowledge and transfer of skills. This is a critical understanding of adult learning and CPD activities, that learning is each person’s responsibility and that it is an active and not a passive process. In addition, CPD is also a dynamic process for healthcare workers that involves proactive engagement, self-directed learning and reflective practice, and that by taking an active approach, they can continually enhance their knowledge and skills. This concurs with the results of a meta-synthesis study of qualitative literature that investigated nurses’ experiences of CPD. It found that those who took responsibility for their learning recognise the importance of lifelong learning and commit to staying updated with the latest advancements, research findings and best practices in their respective fields (Mlambo, Silén & Mcgrath 2021). The meta-synthesis study highlighted that professionals actively pursue their development to uphold high standards of healthcare through competent practices (Mlambo et al. 2021). This was demonstrated by the responses of the participants in this study, indicating their heightened awareness of their responsibility and accountability for the standard of practice and services they provide in their private practice. This is congruent with findings that emphasise that the goal of CPD is to become a lifelong learner. In addition, all healthcare professionals have an ethical obligation to provide up-to-date, high-quality care to patients; however, the literature documents that services could be better served by the private sector than by the public sector (De Wolf & Toebes 2016). The participants stated that participating in CPDs increased their self-confidence, thus increasing self-efficacy, concurring with a case study of healthcare professionals on CPD carried out in the United Kingdom (Manley et al. 2018). Participants in this study further noted that participating in CPD activities was an obligation for them to fulfil their commitment to lifelong learning when entering the profession, which is in keeping with the research findings of a study investigating factors that influence medical practitioners’ CPD in Eswatini (Magwenya & Ross 2021).

A previous research study has highlighted that informal learning opportunities occur through spontaneous encounters with colleagues during interaction courses, allowing for personal introspection based on their colleagues’ clinical encounters (Giri et al. 2012). This is evident in the participant’s responses, as they indicated that they improved their professional development by learning from and reflecting on their peer’s experiences. Interacting with peers allows healthcare workers to receive feedback and support from colleagues who understand their professional context and challenges. Peer feedback can provide valuable insights into areas for improvement, validate achievements and offer encouragement to continue on the CPD journey. Peer support creates a sense of camaraderie and belonging, enhancing motivation and engagement in CPD activities. Peers, who may be older and have more experience, will bring diverse perspectives and experiences to the table, enriching discussions and exposing the young audiologists to different approaches and insights. This diversity fosters critical thinking and encourages young audiologists to consider alternative viewpoints, with peer sharing often involving discussions about real-world cases and challenges encountered in clinical practice. By contextualising learning within practical scenarios, young audiologists can better understand how to apply theoretical knowledge to patient care, and sharing experiences with peers provides validation for healthcare workers’ own experiences and challenges. Additionally, receiving feedback from peers can help identify areas for improvement, validate successful strategies and refine clinical decision-making skills. Encouraging peer interaction and collaboration should be an integral part of CPD programmes to maximise their effectiveness and impact on professional development.

Participants also suggested that their engagement in CPD activities was an educational requirement, as they recognised gaps in their knowledge when managing patients, given the nature of the private healthcare sector and their limited clinical experience. This coincides with the research findings from several studies conducted in low- and middle-income nations, which acknowledged that the private healthcare sector mainly caters to more affluent populations who may have greater expectations from the clinician (Basu et al. 2012). Participants in this study recognised that an important aspect of participating in CPD activities was to keep abreast with the latest innovations. This aligns with a study that highlighted that advances in technology can be expected within audiology when assessing patients, undertaking diagnostics, hearing aid fittings, fine-tuning and counselling (teletherapy), and that engaging with CPD activities will be essential if audiologists are to keep abreast of new developments (Bernstein et al. 2018). Bernstein et al. (2018) further suggested that as a result of the advancements made possible by the internet and smartphones, audiology is evolving, concurring with the responses of the participants from this study. Continuing professional development can assist with equipping young audiologists with the knowledge and skills needed to adapt to these changes and meet the diverse needs of their patients effectively. It can also help young audiologists enhance their clinical decision-making skills and communication skills, leading to greater job satisfaction as well as career advancement.

A study conducted in Australia indicated that an interprofessional, patient-centred approach is essential to manage chronic diseases as well as comorbidities and long-term impairment, such as hearing impairment, which can hinder the daily functioning of patients and negatively affect their overall quality of life (Wei et al. 2020). Externally facilitated interprofessional CPD activities have been reported to increase the professional’s knowledge of their colleague’s scope of practice as well as their social networking, as noted by the participants of this study. This occurs as the health professionals belong to diverse disciplines within the health sector, with collaboration resulting in improved adherence to recommendations and/or referrals addressing the direct needs of the patient (Wei et al. 2020). Participating in CPD activities with healthcare professionals from other disciplines provides opportunities for networking and building professional relationships within the health sector. Building connections with colleagues from diverse disciplines and specialties can open doors to collaboration, mentorship and future learning opportunities. These professional relationships can be valuable sources of support, inspiration and career development throughout the young audiologists’ professional journey and should therefore be considered when planning topics for CPD to allow for the meeting of professionals from different facets of the health sector.

Engaging in CPD activities can boost young audiologists’ confidence in their abilities and knowledge, as seen in the participant’s responses. Practical training sessions, where young audiologists practice and refine their clinical skills under the guidance of experienced professionals, may help young audiologists gain confidence and improve their proficiency. Acquiring new skills and staying updated on advancements within the field will allow young audiologists to become more competent and self-assured clinicians while adapting to the changing healthcare environments. Continuing professional development can be instrumental in supporting the professional development and success of young audiologists and empower them to provide high-quality care, advance their careers and make meaningful contributions to their patient’s quality of life while also ensuring that they are proficient in leveraging technology.

The findings of this study underscored a reoccurring theme among participants regarding their adherence to CPD. While it is known that professional development should be ongoing and address deficits in knowledge and skills, professionals often only comply with CPD because of the mandatory requirements, as seen in the participant’s responses in this study. This is of concern, as this type of engagement with CPD activity rarely leads to improved clinical practice, which is the aim of CPD. It also highlights the deficiency of the current system, which does not promote adult learning but rather takes a ‘tick-box’ approach to acquiring CEU points instead of becoming a lifelong learner. This would concur with an international study in which healthcare professionals were shown to have adopted a ‘tick-box’ mentality when engaging in CPD to ensure adherence to regulatory requirements, thereby offering little relevance for professional or personal development and defeating the purpose of CPD (Mathers et al. 2012).

### Alignment to the conceptual framework

The concepts of andragogy by Malcolm Knowles (1985) provided useful areas around which to develop themes and subthemes. Regarding self-concept, participants indicated a systematic approach to their continual learning process, as they engaged in autonomous learning by choosing CPDs based on their topics of interest. Adults’ previous and current learning experience can impact the way they learn and may influence how they view the need to engage with CPD. This was reflected in the participant’s responses, as they indicated that their adherence to CPD was prompted by specific clinical cases that they were managing or had previously managed. These young audiologists displayed an eagerness to learn where there was a reason to attain new knowledge and wanted to engage in CPD to stay abreast of the most recent developments within their field. Regarding orientation to learning, adults learning should ideally be contextually relevant to address their need, which aligns with their responses, as CPD activities allowed for the transfer of theoretical knowledge into evidence-based practice. Ideally, all CPD activities should be based on adult learning principles and should be problem-centred rather than content-centred learning, encouraging active participation rather than simply meeting the point requirements of the HPCSA.

### Limitations

The audiologists in this study were young, mainly female professionals aged 23 to 26 years, and therefore do not represent the other age groups. In addition, with only 1–5 years of working experience, they had had limited participation in CPD, as they are relatively new graduates. Despite these limitations, the findings provide an overview of areas to be explored in an effort to ensure that audiologist opinions and experiences are taken into account, with a broader age range and experience range as well as more males.

## Conclusion

The findings indicated that participation in CPD improved audiologists’ knowledge and skills while enhancing patient-centred care by acquiring evidence-based practice. Audiologists felt that they had a responsibility to provide the best practices to holistically improve their patient’s health by keeping themselves informed of the latest developments within the field, with CPD being an effective way to acquire new knowledge and skills through both formal and informal interactions. This study underscored the critical importance of considering the experiences and perspectives of young audiologists in the private sector when designing and implementing CPD programmes. Employing the concepts of andragogy can significantly influence CPD engagement among audiologists. CPD programmes tailored to the unique needs and motivations of audiologists will likely be more effective and meaningful. Future CPD initiatives should integrate these insights to foster higher compliance and ensure ongoing professional competency among audiologists. This study not only fills a gap in the existing literature but also provides a foundation for developing more relevant and impactful CPD strategies, ultimately contributing to improved professional standards and patient care in the audiology field.
